# Increased arachidonic acid-containing phosphatidylcholine is associated with reactive microglia and astrocytes in the spinal cord after peripheral nerve injury

**DOI:** 10.1038/srep26427

**Published:** 2016-05-23

**Authors:** Dongmin Xu, Takao Omura, Noritaka Masaki, Hideyuki Arima, Tomohiro Banno, Ayako Okamoto, Mitsuru Hanada, Shiro Takei, Shoko Matsushita, Eiji Sugiyama, Mitsutoshi Setou, Yukihiro Matsuyama

**Affiliations:** 1 Department of Orthopaedic Surgery, Hamamatsu University School of Medicine, 1-20-1, Handayama, Higashi-ku, Hamamatsu, Shizuoka 431-3192, Japan; 2 Department of Cell Biology and Anatomy, Hamamatsu University School of Medicine, 1-20-1, Handayama, Higashi-ku, Hamamatsu, Shizuoka 431-3192, Japan; 3 Department of Systems Molecular Anatomy, Institute for Medical Photonics Research, Preeminent Medical Photonics Education & Research Center, Hamamatsu University School of Medicine, 1-20-1, Handayama, Higashi-ku, Hamamatsu, Shizuoka 431-3192, Japan; 4 The Institute of Medical Science, The University of Tokyo, 4-6-1 Shirokanedai, Minato-ku, Tokyo 108-8639, Japan; 5 Department of Anatomy, The University of Hong Kong, Pokfulam, Hong Kong, 999077 China; 6 Division of Neural Systematics, National Institute for Physiological Sciences, 38 Nishigonaka Myodaiji, Okazaki, Aichi, 444-8585, Japan

## Abstract

Peripheral nerve injury (PNI) triggers cellular and molecular changes in the spinal cord. However, little is known about how the polyunsaturated fatty acid-containing phosphatidylcholines (PUFA-PCs) are regulated in the spinal cord after PNI and the association of PUFA-PCs with the non-neuronal cells within in the central nervous system (CNS). In this study, we found that arachidonic acid-containing phosphatidylcholine (AA-PC), [PC(16:0/20:4)+K]^+^, was significantly increased in the ipsilateral ventral and dorsal horns of the spinal cord after sciatic nerve transection, and the increased expression of [PC(16:0/20:4)+K]^+^ spatiotemporally resembled the increase of reactive microglia and the astrocytes. From the lipidomics point of view, we conclude that [PC(16:0/20:4)+K]^+^ could be the main phospholipid in the spinal cord influenced by PNI, and the regulation of specific phospholipid molecule in the CNS after PNI is associated with the reactive microglia and astrocytes.

Peripheral nerve injury (PNI) provokes changes in neuronal, glial and immuno interactions within the spinal cord. Although the alteration of neuropeptides, proteins and transcription factors in the spinal cord following spinal nerve transection (SNT) have been extensively studied in recent years^1^,^2^,^3^, changes in glycerophospholipids in the spinal cord after PNI remains unknown. Glycerophospholipids consist of four primary phospholipids; phosphatidylcholine (PC), phosphatidylethanolamine, phosphatidylserine, and phosphatidylinositol. Among the phospholipids, PC is the most abundant accounting for >50% of the phospholipids, and influences cellular function^4^,^5^. Polyunsaturated fatty acids (PUFA), such as arachidonic acid (AA) and docosahexaenoic acid (DHA), are considered to be involved in neuronal injury. Indeed, AA and its derivatives, prostaglandins, have been confirmed to be involved in neuro-inflammatory processes and neuropathic pain^6^,^7^. DHA is known to exhibit neuronal protective effects after spinal cord injury (SCI)^8^. Despite the important role of PUFAs in the nervous system, little is known about their main precursors, polyunsaturated fatty acid-containing phosphatidylcholines (PUFA-PCs), in the mechanism of neuronal injury. Recently, imaging mass spectrometry (IMS) has emerged as a powerful method that has promoted lipid research into a new phase where membrane lipids can be visualized and detected in specific regions of the various substances^9^,^10^,^11^. Previous studies utilizing matrix-assisted laser desorption/ionization imaging mass spectrometry (MALDI-IMS) revealed that the characteristic alterations of arachidonic acid-containing phosphatidylcholines (AA-PCs) after SCI were associated with immune response^12^. In addition, the docosahexaenoic acid-containing phosphatidylcholines (DHA-PCs) are considered to exert neuroprotective effects after spinal contusion^13^. Although these studies showed that AA-PCs and DHA-PCs, as well as their derivatives play increasingly important roles in the process of neuronal injury, the spatiotemporal changes of PUFA-PCs in the spinal cord after PNI are unknown. Microglia and astrocytes, which are the main glial cells in the central nervous system (CNS), are rapidly activated after PNI^14^,^15^. Microglial cells are the resident macrophages in the CNS and can be activated via phosphorylation of p38 MAP kinase after PNI^16^. Activation of astrocytes in the spinal cord is known to provoke bilateral allodynia after PNI^17^. Moreover, studies have revealed that microglia and astrocytes are capable of releasing arachidonic acid^18^,^19^. Although the activity of glial cells after PNI in the spinal cord has been extensively studied, the association between PUFA-PCs and the glial cells remain unknown. In order to clarify these questions, we investigated the changes of AA-PCs, DHA-PCs in relation to the glial cells in the spinal cord following SNT using MALDI-IMS and immunohistochemistry. The distribution of AA-PCs and DHA-PCs were investigated in positive ion mode of MALDI-time of flight (TOF) mass spectrometry.

## Results

### Comparison of mass spectra of ventral and dorsal horns of sham and SNT mice

**Figure 1:**
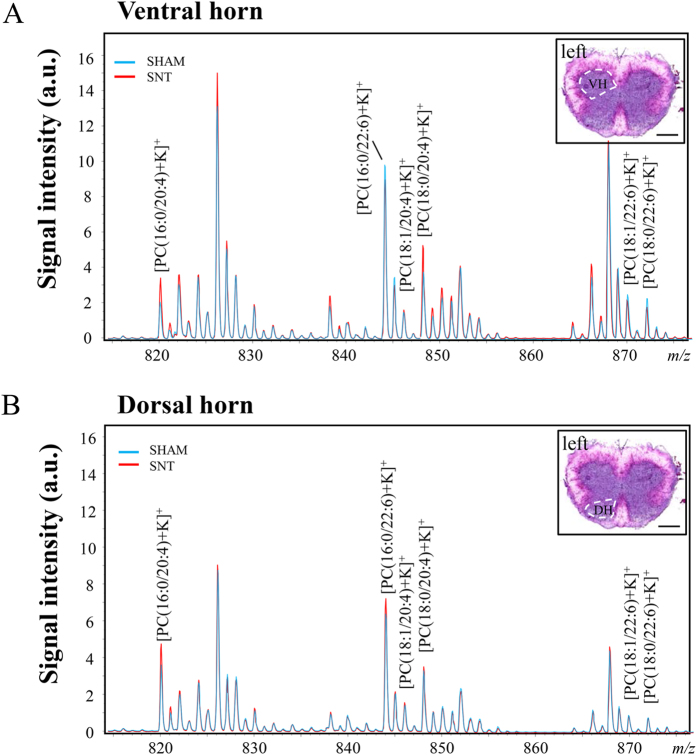
Comparison of mass spectra of PUFA-PCs in sham and SNT mice spinal cords, 7 days after surgery. (**A**) Comparison of mass spectra of PUFA-PCs in ventral horn between sham (blue) and SNT (red). (**B**) Comparison of mass spectra of phospholipids in the dorsal horn in sham and SNT. AA-PCs: [PC(16:0/20:4)+K]^+^ (*m*/*z* 820.5), [PC(18:1/20:4)+K]^+^ (*m*/*z* 846.5), and [PC(18:0/20:4)+K]^+^ (*m*/*z* 848.5) and DHA-PCs: ([PC(16:0/22:6)+K]^+^ (*m*/*z* 844.5), [PC(18:1/22:6)+K]^+^ (*m*/*z* 870.5), and [PC(18:0/22:6)+K]^+^ (*m*/*z* 872.5) shown in A and B were the target phospholipids in the present study. Insets are the HE staining of mouse spinal cord illustrating the region of interest, ventral horn in (**A**), dorsal horn in (**B**). VH, ventral horn; DH, dorsal horn. Scale bar: 500 μm.

We first examined the lipid composition of the spinal cord 7 days following SNT. The comparison of ion abundances between the sham and the SNT group was performed on two ROIs: ventral (Fig. 1A) and dorsal (Fig. 1B) horns of the spinal cord. Mass spectra ranging from *m*/*z* 500 to 1000 were collected. Focusing on the range from *m*/*z* 800 to 880, six target phospholipids identified in the previous study were analyzed^12^; AA-PCs: [PC(16:0/20:4)+K]^+^ (*m*/*z* 820.5), [PC(18:1/20:4)+K]^+^ (*m*/*z* 846.5), and [PC(18:0/20:4)+K]^+^ (*m*/*z* 848.5), and DHA-PCs: [PC(16:0/22:6)+K]^+^ (*m*/*z* 844.5), [PC(18:1/22:6)+K]^+^ (*m*/*z* 870.5), and [PC(18:0/22:6)+K]^+^ (*m*/*z* 872.5). These PCs were selected in our study for their known association with the CNS^9^,^12^,^20^(Fig. 1).

### Increased expression of [PC(16:0/20:4)+K]^+^ in ventral and dorsal horn of mouse spinal cord 7 days after SNT

**Figure 2:**
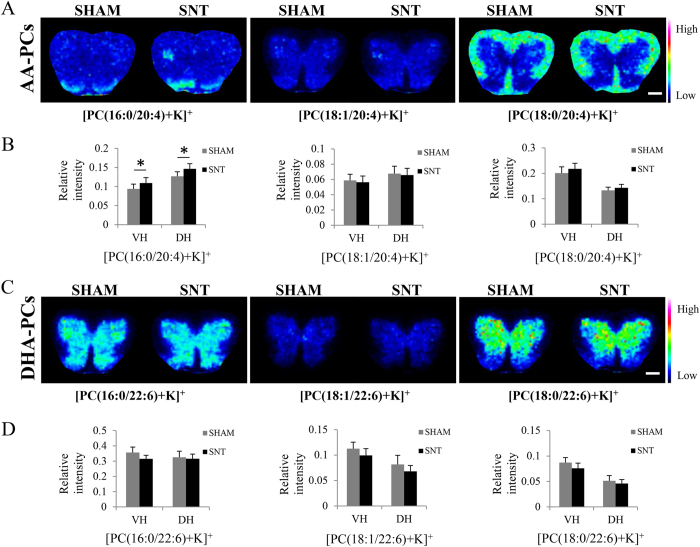
Distribution of PUFA-PCs in spinal cord of sham and SNT mice 7 days after injury. (**A**) Distribution of AA-PCs in the spinal cord. [PC(16:0/20:4)+K]^+^ was increased in the ipsilateral ventral and dorsal horns of SNT. (**C**) The distribution of DHA-PCs in the spinal cord. (**B**,**D**) are the quantitative comparisons of the six PUFA-PCs between sham and SNT (n = 7). *p < 0.05 indicates a significant difference by Student’s *t*-test. Scale bar: 500 μm.

**Figure 3:**
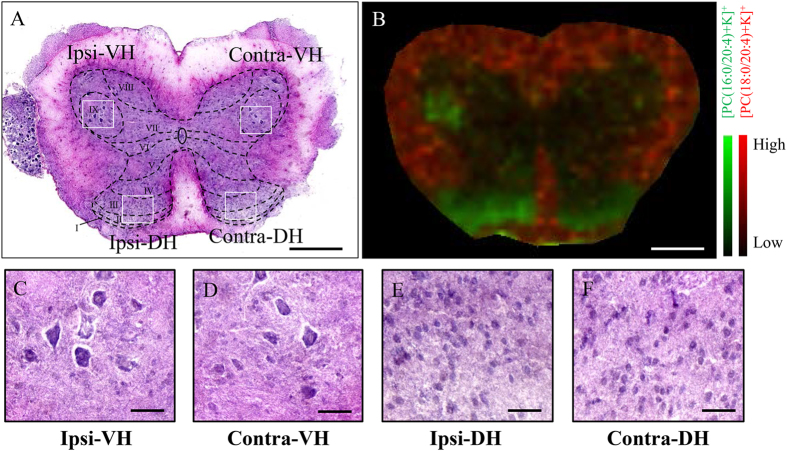
[PC(16:0/20:4)+K]^+^ was increased in laminae I-III of the dorsal horn and laminae IX region of the ventral horn. (**A**) Schematics of spinal laminae are included in an HE image of a coronal section of the lumbar spinal cord. (**B**) Merged image of [PC(16:0/20:4)+K]^+^ (green) and [PC(18:0/20:4)+K]^+^ (red) demonstrated that [PC(16:0/20:4)+K]^+^ was increased in laminae IX region of the ventral horn and laminae I–III of the dorsal horn. (**C–F**) Magnified images showed that no cellular infiltration occurred in the ipsilateral side in comparison with contralateral side after SNT. Scale bar: 500 μm in (**A**,**B**); 100 μm in (**C**–**F**).

We then visualized these six phospholipids of interest using MALDI-IMS. In the sham mice, the expression of each phospholipid displayed distinct patterns in the spinal cord: [PC(16:0/20:4)+K]^+^ was highly expressed in the dorsal horn; [PC(18:1/20:4)+K]^+^, [PC(16:0/22:6)+K]^+^, [PC(18:1/22:6)+K]^+^, and [PC(18:0/22:6)+K]^+^ were found to be expressed in the gray matter and [PC(18:0/20:4)+K]^+^ was enriched in the white matter (Fig. 2A,C). Interestingly, we found that [PC(16:0/20:4)+K]^+^ was increased in both the ipsilateral ventral and dorsal horns 7 days after SNT. We then quantified the expression levels of each phospholipid within the ventral and dorsal horns (7 mice in each group). Analysis revealed a significant increase of [PC(16:0/20:4)+K]^+^ both in the ventral (p = 0.001) and dorsal (p = 0.011) horns (Fig. 2B,D). In addition, we found that [PC(16:0/20:4)+K]^+^ was specifically increased in the gray matter in laminae I-III of the dorsal horn and laminae IX of the ventral horn (Fig. 3A,B).

### Increased microglia and astrocytes in the region where [PC(16:0/20:4)+K]^+^ was highly expressed 7 day after SNT

**Figure 4:**
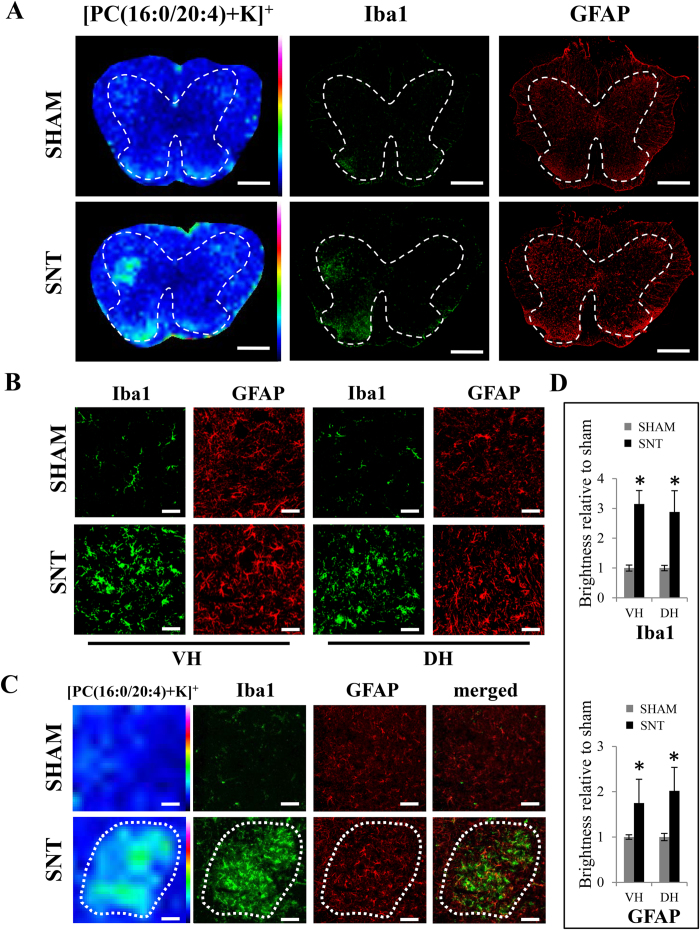
Increased levels of Iba1 and GFAP-positive cells located in the [PC(16:0/20:4)+K]^+^ highly expressing region 7 days after SNT. (**A**) Distribution of [PC(16:0/20:4)+K]^+^ and consecutive sections stained with Iba1 and GFAP antibodies. Dashes represent the spinal cord gray matter. (**B**) Immunostaining of Iba1 and GFAP in the ventral and dorsal horns between sham and SNT mice. (**C**) Similar area of increased expression [PC(16:0/20:4)+K]^+^, Iba1 and GFAP. (**D**) Quantitative analysis of Iba1 and GFAP immunofluorescence intensity. *p < 0.05 indicates a significant difference between sham and SNT mice (n = 7). Scale bar: 500 μm in (**A**), 50 μm for the magnified images in (**B**), and 100 μm in (**C**).

**Figure 5:**
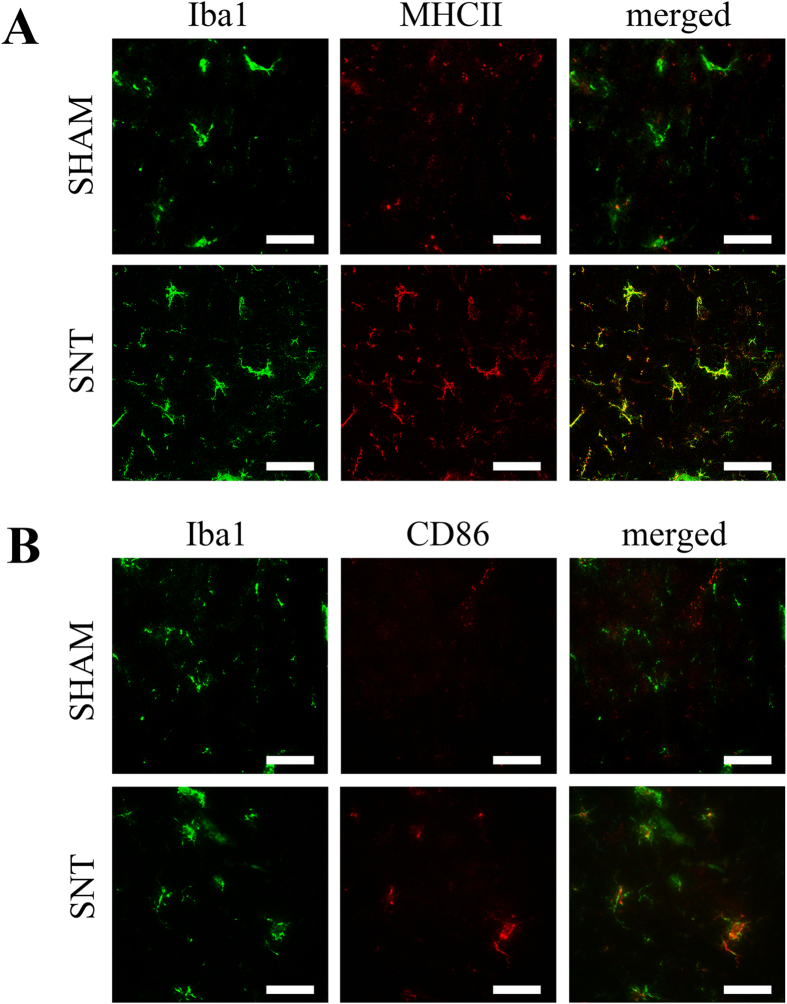
Microglial cells were activated after SNT. (**A**) Iba1 positive microglia were co-labeled with activated microglia marker MHCII in the spinal cord after SNT. (**B**) Activated microglia marker CD86 was found in Iba1 positive microglia in the spinal cord after SNT. Scale bar: 50 μm.

Microglia expression in the spinal cord is known to increase after PNI as a central immune response or as a pain mediator^14^,^21^,^22^. Moreover, SNT also results in increased expression of astrocytes in the ipsilateral spinal cord^23^. Therefore, we used anti-Iba1 and anti-GFAP antibodies to analyze distributions of microglia and astrocytes in the spinal cord after SNT (n = 7 per group). We found that the immunofluorescence intensity of Iba1 positive cells was significantly higher in the SNT mice than in the sham (p = 0.001 in ventral horn, p = 0.001 in dorsal horn) (Fig. 4B,D). The increased microglia observed in the spinal cord suggested the activation of these cells after SNT. To address this question, we performed Iba1, MHCII and CD86 staining (Fig. 5A,B) to label both activated and non-activated microglia. We found that after SNT, Iba1 positive microglia also became immuno-positive for MHCII and CD86 staining, confirming the activation of these cells (Fig. 5). In addition, the immunoreactivity of GFAP was significantly increased in the ventral and dorsal horns where Iba1 positive cells and [PC(16:0/20:4)+K]^+^ were highly expressed (p = 0.003 in ventral horn, p = 0.019 in dorsal horn) (Fig. 4B,D). Interestingly, the locations of Iba1 positive microglia and GFAP positive astrocytes resembled the areas highly expressing [PC(16:0/20:4)+K]^+^ (Fig. 4C).

### Similar spatiotemporal alteration patterns of microglia and astrocytes with [PC(16:0/20:4)+K]^+^

**Figure 6:**
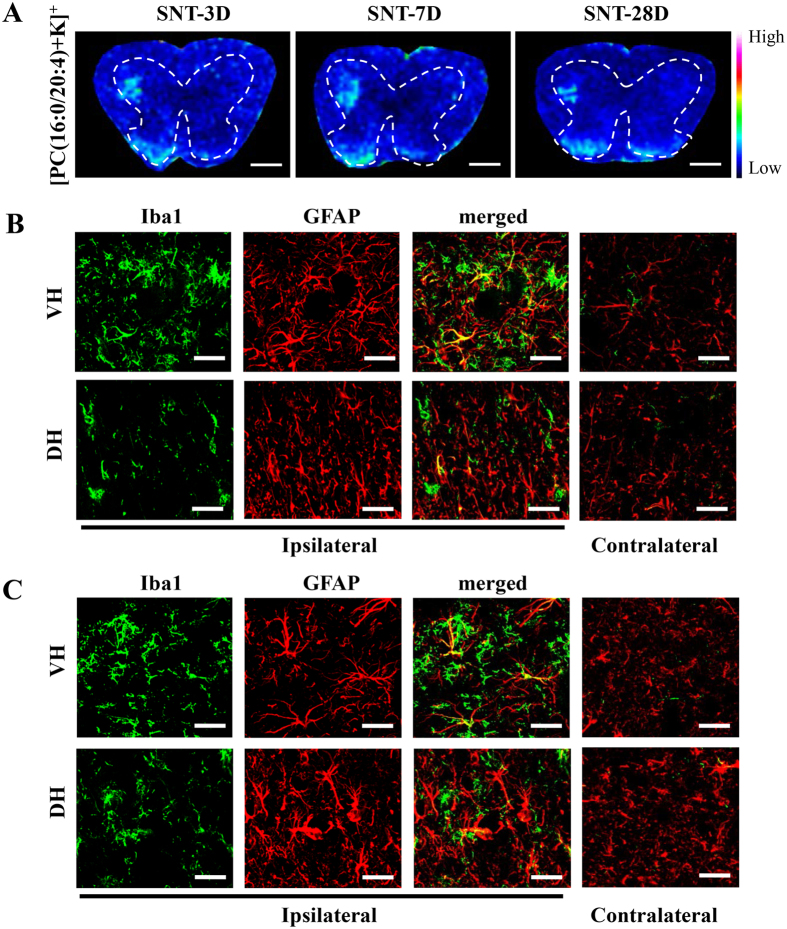
Sustained [PC(16:0/20:4)+K]^+^ expression in the spinal cord up to 4 weeks after SNT. (**A**) The distribution of [PC(16:0/20:4)+K]^+^ in spinal cord at 3, 7 and 28 days after SNT. Dashes represent the spinal cord gray matter. (**B**) Immunohistological images for Iba1 and GFAP of VH and DH 3 days after SNT. (**C**) Images for Iba1 and GFAP of VH and DH 28 days after SNT. Scale bar: 500 μm in (**A**), 50 μm in (**B**,**C**).

In the spinal cord, microglia become activated immediately after SNT, and are sustained for several weeks with a slow decrease^24^. Moreover, compared with the microglia, the astrocyte response to the PNI is delayed and persists for a long time^25^. Thus, we additionally analyzed the spinal cord on days 3 and 28 days post SNT. As expected, high immunoreactivities of Iba1 and GFAP were observed in the ventral and dorsal horns from day 3 to 28 together with elevated expression of [PC(16:0/20:4)+K]^+^ (Fig. 6).

### Preservation of DHA-PCs in the spinal cord 28 days after SNT

**Figure 7:**
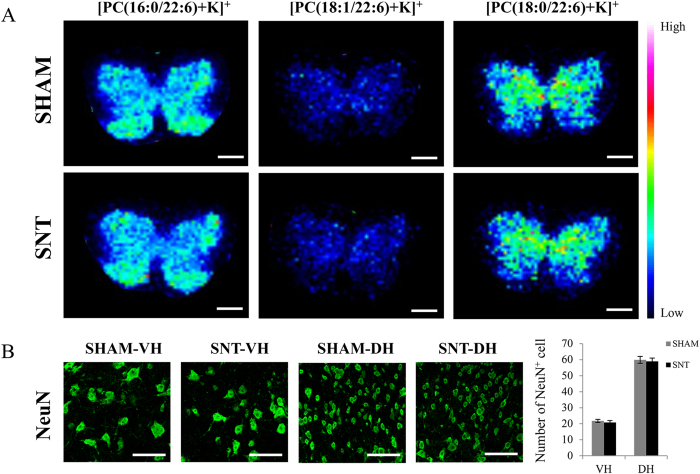
No significant changes in DHA-PCs were observed in the spinal cord at 28 days after SNT. (**A**) Ion images of [PC(16:0/22:6)+K]^+^, [PC(18:1/22:6)+K]^+^, and [PC(18:0/22:6)+K]^+^ representing DHA-PCs. These ions showed no significant changes between sham and SNT at 28 days after surgery. (**B**) Images of NeuN^+^ neurons in the ventral and dorsal horns of sham and SNT. Quantification of the NeuN^+^ neurons showed no significant difference between the two groups (n = 4 per group). Scale bar: 500 μm in (**A**), 100 μm in (**B**).

We also evaluated the expression of DHA-PCs ([PC(16:0/22:6)+K]^+^, [PC(18:1/22:6)+K]^+^ and [PC(18:0/22:6)+K]^+^) at 28 days post SNT. Compared with notably increased expression of [PC(16:0/20:4)+K]^+^ in the spinal cord, none of the DHA-PCs showed any changes after SNT. Due to the close relationship between DHA-PCs and the neurons, we also analyzed the degenerative changes of the neurons via the quantitative analysis of NeuN positive cells. We found no significant neuronal loss in the spinal cord 28 days after SNT (4 mice in each group, p = 0.518 in ventral horn, p = 0.625 in dorsal horn) (Fig. 7).

## Discussion

PNI results in complex changes of neuronal networks, glia and immune cell interactions and the regulation of neuropeptides, proteins and transcription factors in the spinal cord. In the present study, we focused on analyzing spatial distribution of the PUFA-PCs containing AA-PCs and DHA-PCs in the spinal cord after SNT using MALDI-IMS. The most notable finding in the present study was that [PC(16:0/20:4)+K]^+^ was increased both in ventral and dorsal horns, and was associated with the activation of microglia and astrocytes after SNT.

AA is known to be released into the cytoplasm from the cellular membrane phospholipids with the activation of phospholipase A2, and it can be further transformed into a variety of derivatives which participate in inflammatory reactions^26^. Prostaglandin E2 is well known to contribute to the maintenance of neuropathicpain^27^. Although in this present study, we could not reveal direct evidence of increased prostaglandin E2 or other AA derivatives, [PC(16:0/20:4)+K]^+^ which was continuously expressed after SNT, could be the source contributing to the elevation of AA and its derivatives.

PUFA-PCs, which are the main precursors of PUFA, are associated with cell types, and influence the properties of membranes^9^,^28^,^29^. Microglia and astrocytes, which are the main glial cells in the CNS, immediately react to PNI. Spinal microglial cells, which are the resident mononuclear phagocytes of the CNS, are known to be activated after PNI via different signaling pathways, such as colony-stimulating factor 1^30^. The interesting finding of our study was that a similar spatiotemporal expression pattern was observed between [PC(16:0/20:4)+K]^+^ and activated microglial cells. Microglial cells are morphologically and functionally dynamic cells which have different morphological phenotypes^31^. Environmental alterations induce morphological changes in microglia^32^. Cellular membranes are constructed by lipids, especially the rich-content phospholipids which are required for shape changes. Taking into account the simultaneous increase of [PC(16:0/20:4)+K]^+^ and the reactive microglia, we speculated that [PC(16:0/20:4)+K]^+^ may be associated with reactive microglia via increasing AA-PC composition in the microglial membrane.

Activation of astrocytes in the dorsal and ventral horn in response to PNI is a response just as important as the activation of microglia^23^,^33^. Similar to microglia cells, astrocytes also have reactive and non-reactive conditions, which can be assessed by a different morphological appearance and by increased GFAP staining^34^,^35^. Recent studies have shown that astrocytes can be activated via p38 Mitogen-Activated Protein Kinase^33^ or by interleukin-18 released from activated microglia^36^. Interestingly, extracellular addition of AA induces release of AA from astrocytes^37^. In this present study, we also found the reactive astrocytes occurred in the region of increased [PC(16:0/20:4)+K]^+^ expression and microglia activation. Together with our studies, we hypothesize that the activation of microglia resulted in the elevation of [PC(16:0/20:4)+K]^+^ and the activation of astrocytes, which further led to the increase in [PC(16:0/20:4)+K]^+^.

In our spatiotemporal study, we observed increased microglia, astrocytes and the expression of [PC(16:0/20:4)+K]^+^ persisting until 28 days after SNT, which could be reflecting the development and maintenance of neuropathic pain^38^,^39^, or neurodegeneration^40^.

DHA is a major PUFA in the CNS, and exerts its neuroprotective role through the M2 phenotype towards microglial polarization^41^,^42^. DHA-containing phospholipids can be catalyzed by free radicals and generate lipid peroxides, such as F4-isoprostanes that are associated with neurodegenerative diseases^43^. As indispensable membrane phospholipids, DHA-PCs are required in motor neurons^12^. [PC(16:0/22:6)+K]^+^ was found to be enriched in the large motor neurons of the ventral horn of the spinal cord, and was dramatically reduced one day after SCI^12^. However in our study, neither DHA-PCs nor NeuN positive cell showed significant change in the spinal cord 28 days after SNT. Two studies observed degenerative changes of neurons in the ventral and dorsal horns within one month after PNI using rat models^44^,^45^. Therefore, species differences could account for the preserved neuron number in the mouse spinal cord.

Although MALDI-IMS is an advanced technique to visualize and analyze the lipid molecules, it still has limitations with respect to spatial resolution^46^. Therefore, it was technically impossible to identify the exact cell responsible for the increased elevation of [PC(16:0/20:4)+K]^+^ and to perform *in vitro* single cell analysis. This remains to be addressed in our future studies. We conclude that AA-PC could be the main phospholipid reflecting reactive microglia and astrocytes in the spinal cord after PNI and further investigation is required to identify the exact cell sources.

## Methods

### Animals

8-week-old C57BL/6JJmsSlc female mice (16–21 g) purchased from SLC Inc. (Hamamatsu, Japan) were used for this study. All experimental protocols were performed in accordance with the guidelines by the Ethics Committee of Hamamatsu University School of Medicine. All experiments were conducted according to protocols approved by the Animal Care and Use Committee of the Hamamatsu University School of Medicine.

### SNT

Mice were deeply anesthetized with 40 mg/kg pentobarbital sodium. SNT was performed by exposing the left sciatic nerve at mid-thigh level, ligating with 6–0 suture, and transecting distal to the ligature. The muscle and skin layers were closed by nylon sutures. Sham-operated mice underwent nerve exposure without ligation and transection of the sciatic nerve. All the mice were housed in a colony room controlled for temperature and humidity with a 12:12-h light/dark cycle (lights on at 08:00 h) with food and water available ad libitum.

### Tissue section preparation

Three, 7 and 28 days post-operatively, mice were anesthetized with 40 mg/kg pentobarbital sodium and euthanized by transcardial perfusion with cold phosphate buffered saline (PBS). L3-5 lumbar spinal cord segments were harvested and immediately embedded in a pre-cooled solution of 2% carboxymethyl cellulose (CMC) sodium salt (Wako, Osaka, Japan), and then frozen in powdered dry ice. The solid tissues embedded in CMC were stored at −80 °C until sectioning. Tissues were sliced into 10 μm thick axial serial sections with a cryostat (CM1950; Leica, Wetzler, Germany). Tissue sections from sham and SNT mice were mounted onto the same indium-tin-oxide (ITO)-coated glass slides (Bruker Daltonics, Billerica, MA, USA) for MALDI-IMS analysis, and consecutive sections of those were also mounted onto the Matsunami Adhesive Silane (MAS)-coated glass slide (Matsunami, Osaka, Japan) for immunohistochemistry. All the sections were stored at −80 °C until matrix application.

### MALDI-IMS

Samples on ITO-coated glass slides were sprayed with 1 mL matrix solution (40 mg/mL 2,5-dihydroxybenzoic acid), 20 mM potassium acetate, 70% methanol) (Bruker Daltonics) using a 0.2 mm nozzle caliber airbrush (Procon Boy FWA Platinum; Mr. Hobby, Tokyo, Japan). Distance between the nozzle tip and the slice surface was kept at 10 cm, and spraying was performed for 15 min for uniform matrix deposition. Positive ions of the spinal cord were detected using a MALDI-TOF/TOF-type instrument (ultraflex II TOF/TOF;Bruker Daltonics) equipped with a 355 nm Nd:YAG laser. The laser was set to the minimum spot size with 20% laser power. Mass spectra ranging from mass-to-charge ratio (*m*/*z*) 500 to 1000were collected. Laser scan pitch was set to 50 μm. The calibration of *m*/*z* values was performed for each IMS measurement using calibration standard substances: 2,5-dihydroxybenzoic acid, bradykinin (Sigma-Aldrich, St Louis, MO, USA), and angiotensin II (Sigma-Aldrich). Regions of interest (ROIs) in spinal cord were determined by comparison with the HE staining result from consecutive tissue sections. Signals were collected using flexControl software (Bruker Daltonics) and reconstruction of ion images (normalized by total ion current) was performed with flexImaging4.0 software (Bruker Daltonics).

### Immunohistochemistry

Frozen sections mounted on MAS-coated glass slides were equilibrated at room temperature for 10 min, and then fixed with 2% paraformaldehyde for 15 min at room temperature. After washing three times with PBS for 5 min, sections were blocked with blocking solution (PBS containing 1% bovine serum albumin, 2% blocking reagent, and 0.1% Triton X-100) for 60 min at room temperature. The primary antibodies: rabbit anti-Iba1 (1:500; Wako, Osaka, Japan), chicken anti-GFAP (1:1000;Abcam, Cambridge, United Kingdom), Rabbit anti-NeuN antibody (1:500; Millipore, Billerica, 213 MA, USA), anti-MHC Class II antibody (1:100; Abcam, Cambridge, United Kingdom) and rat anti-mouse CD86 (1:200; BD Biosciences, San Jose, CA, USA) were conjugated in a humidified chamber at 4 °C overnight, and then washed three times with PBS and incubated for 60 min at room temperature with secondary antibody solution: Alexa Fluor 488 donkey anti-rabbit IgG (Life Technologies, Carlsbad, CA, USA), Alexa Fluor 594 goat anti-chicken IgG (Life Technologies, Carlsbad, CA, USA) or Alexa Fluor594 goat anti-mouse IgG (Abcam, Cambridge, United Kingdom).The slides were mounted with VECTASHIELD Antifade Mounting Medium with DAPI (Vector Laboratories, Inc, Burlingame, USA). Images were acquired using an automated image scanner (NanoZoomer 2.0HT; Hamamatsu Photonics, Shizuoka, Japan) and confocal microscope (FV1000-D, OLYMPUS, Tokyo, Japan).

### Immunohistological quantification

Immunohistological images were processed using the free image analysis software ImageJ 1.49 m (NIH, MD, USA) to measure the mean density of Iba1- or GFAP-immunoreactivity after subtracting background grey levels. For comparison between the injured and uninjured, immunofluorescence intensity was quantified within a fixed area of the central substantia gelatinosa (0.25 mm^2^) and the ratios of ipsilateral to contralateral sides were analyzed for each animal. For the comparison within the injury side, the ratios of ventral and dorsal horns to the central canal were evaluated according to same size regions in the ventral horn, dorsal horn, and central canal.

### Cell count procedures

The lumbar spinal cords from 8 mice (4 mice each for sham and SNT) were sliced into 20 μm thick axial serial sections with a cryostat (CM1950; Leica, Wetzler, Germany). Neurons localized in the ventral and dorsal horns were counted in every fifth section (5 sections/animal in each group). The number of NeuN positive cells were counted within a 300 μm × 300 μm field in the VH and 200 μm × 200 μm field in the DH using ImageJ 1.49 m (NIH, MD, USA) for each animal.

### Statistical analysis

All quantitative data are presented as mean ± SEM. The statistical significance of difference between values was determined using paired Student’s t-tests and one-way ANOVA followed by post hoc tests. The differences were considered as significant when the p value was <0.05. Statistical analysis was performed using the Statistical Package for the Social Science (SPSS) software (version 18; SPSS, Chicago, IL, USA).

## Additional Information

**How to cite this article**: Xu, D. *et al*. Increased arachidonic acid-containing phosphatidylcholine is associated with reactive microglia and astrocytes in the spinal cord after peripheral nerve injury. *Sci. Rep.* **6**, 26427; doi: 10.1038/srep26427 (2016).
